# Effectiveness of neuromuscular electrical stimulation for endometriosis-related pain: A protocol of systematic review and meta-analysis

**DOI:** 10.1097/MD.0000000000020483

**Published:** 2020-06-19

**Authors:** Zi-yang Zhang, Jing Wang, Yi-lin Fan, Bi-yao Wang, Wei-ting Zhang

**Affiliations:** a Department of Gynecology; b Department of Physical Diagnostics; c Department of Nuclear Medicine; d Department of Blood Transfusion; e Department of Internal Medicine, Affiliated Hongqi Hospital of Mudanjiang Medical University, Mudanjiang, China.

**Keywords:** effectiveness, endometriosis-related pain, neuromuscular electrical stimulation, safety

## Abstract

**Background:**

This study will assess the effectiveness and safety of neuromuscular electrical stimulation (NMES) for endometriosis-related pain (ERP).

**Methods:**

Seven electronic databases of Cochrane Library, PUBMED, EMBASE, WANGFANG, VIP, CBM, and CNKI will be searched. We will search all electronic databases related the randomized controlled trials (RCTs) on the effectiveness and safety of NMES for ERP up to the March 31, 2020 without restrictions of language. RevMan 5.3 software will be used for risk of bias assessment, related data analysis and meta-analysis.

**Results:**

This systematic review and meta-analysis will summarize current high-quality RCTs on the effectiveness and safety of NMES for ERP. Results of this study will provide the basis for both clinician and further research.

**Conclusion:**

This study will investigate whether NMES is effective and safety for the treatment of ERP.

**Systematic review registration:**

INPLASY202040191.

## 1 Introduction

Endometriosis is a chronic inflammatory disease in reproductive-age females.^[1,2]^ It is estimated that it affects 5% to 15% female population,^[3,4]^ and manifests as dysmenorrheal, and pelvic pain (also known as endometriosis-related pain (ERP)), which often result in poor quality of life in such patients.^[5–7]^ Current pain managements involve a variety of pharmacological and surgical treatments.^[8–11]^ However, most of them have insufficient efficacy and considerable side effects.^[8–11]^ Thus, neuromuscular electrical stimulation (NMES) may sever as an alternative treatment for ERP relief. Although several studies reported the effectiveness and safety of NMES for ERP,^[12–19]^ no systematic review has addressed this topic. The purpose of this systematic review is to judge whether NMES is effective and safety in treating ERP.

## 2 Methods

### 2.1 Study registration

This study was registered and funded on INPLASY202040191. It has been reported based on the guidelines of the Preferred Reporting Items for Systematic Reviews and Meta-Analysis Protocol statement.^[20,21]^

### 2.2 Inclusion criteria for study selection

#### 2.2.1 Type of study

The randomized controlled trials (RCTs) of NMES for ERP will be included in this study. However, non-RCTs and quasi-RCTs will be excluded.

#### 2.2.2 Type of patients

The patients who diagnosed with ERP will be included with no restriction of race and age. All patients who had heart disease (e.g., measured by electrocardiogram test) without NMES therapy will be excluded in this study.

#### 2.2.3 Type of interventions

Experimental group: The studies in which the experimental group receiving NMES will be included with no limitations of dosage, duration, and frequency.

Control group: Patients in the control group can be administered any therapies, except NMES.

#### 2.2.4 Types of outcome measurements

The primary outcome is pelvic pain intensity, as measured by Numeric Rating Scale or relevant scales.

The secondary outcomes include dyspareunia, patient satisfaction, quality of life, electrocardiogram test, and adverse effects.

### 2.3 Search methods for identification of studies

#### 2.3.1 Electronic databases

Seven electronic bibliographic databases including PUBMED, Cochrane Library, EMBASE, WANGFANG, VIP, CBM, and CNKI will be retrieved. All databases will be searched related the RCTs on the effectiveness and safety of NMES for ERP up to the March 31, 2020 without restrictions of language. The search strategy sample for PUBMED is presented in Table 1. Similar search strategies for other electronic databases will be applied.

**Table 1:**
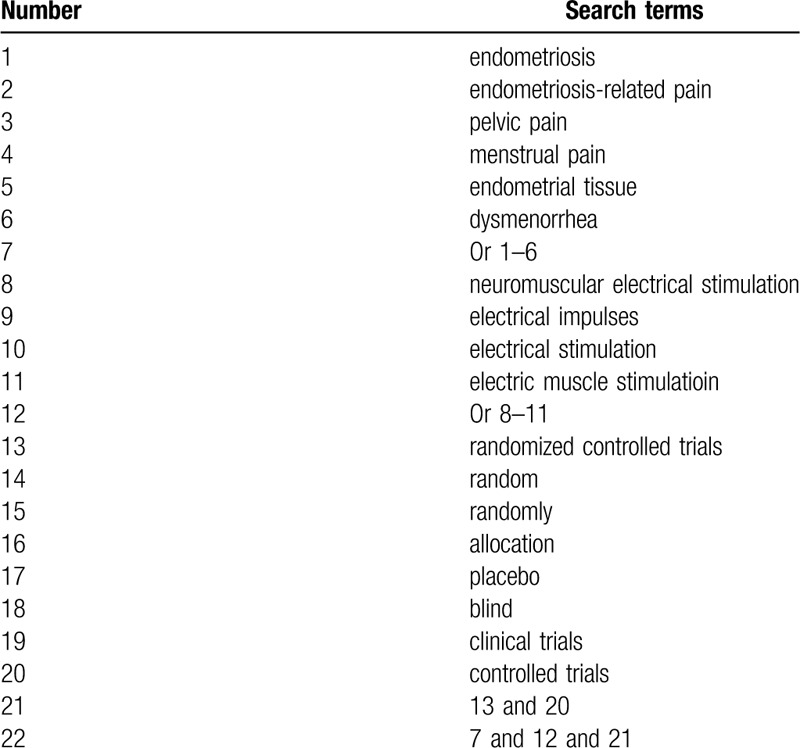
Search strategy for PUBMED.

#### 2.3.2 Searching other resources

We will also search dissertations, clinical registry, and reference lists of relevant studies to avoid missing any potential studies.

### 2.4 Data collection and analysis

#### 2.4.1 Study selection

Two reviewers will independently scan the titles and abstracts of all searched literatures. Then, the irrelevant and duplicated studies will be removed. After that, we will further check full-text of remaining studies for the eligibility. Any divergences will be eliminated through discussion or consulting a third reviewer. The procedure of study selection is illustrated in a flow diagram.

#### 2.4.2 Data collection and management

Two reviewers will independently collect data from each included trial using a pre-designed data extraction form. A third reviewer will help to solve different opinions between 2 reviewers regarding the data collection. We will extract the following related data: general information (title, first author, publication time, and location), patient information (race, age, inclusion, and exclusion criteria), study methods (sample size, randomization, blinding, and allocation), treatment details (dosage, frequency, duration), outcome measurements (all outcome measurements and safety), and other detailed information. If there is missing information, we will contact corresponding author from each primary study to obtain it.

#### 2.4.3 Assessment of risk of bias of included studies

Two reviewers will independently assess the risk of bias for each included study using Cochrane risk of bias. It includes selection bias, performance and detection bias, attrition bias, reporting bias, and other bias. Furthermore, each 1 is further classified as low, unclear and high risk of bias. If there are any divergences, an arbiter will solve them via discussion.

#### 2.4.4 Detection of treatment effect

Dichotomous values will be employed as risk ratio and 95% confidence intervals (CIs), while the continuous values will be expressed as mean difference or standardized mean difference and 95% CIs.

#### 2.4.5 Assessment of heterogeneity

*I*^2^ statistics will be used to assess the heterogeneity among included studies in this study. The value of *I*^2^ ≤ 50% is regarded as reasonable heterogeneity; and the value of *I*^2^ > 50% is considered as having significant heterogeneity.

#### 2.4.6 Subgroup analysis

Subgroup analysis will be performed in accordance with the various interventions, comparators, and outcomes if these data are available.

#### 2.4.7 Sensitivity analysis

The sensitivity analysis will be carried out to check the robustness and stability of pooled outcomes by removing studies with low quality, insufficient information, and different statistical models.

#### 2.4.8 Publication bias

If there are more than 10 eligible RCTs in this study, we will check potential reporting bias using Funnel plot and Egger regression test.^[22]^

#### 2.4.9 Data synthesis

All statistical analysis will be carried out using RevMan 5.3 software. We will perform meta-analysis if there are sufficient studies (2 or more) based on the same treatments and outcome measurements. If there is reasonable heterogeneity among included studies, we will use a fixed-effects model to pool the data. If there is significant heterogeneity among eligible studies, we will utilize a random-effects model to synthesize the data. At the same time, we will employ subgroup analysis and meta-regression test to identify the sources of substantial heterogeneity.

### 2.5 Dissemination and ethics

This study will not need ethical approval, because no individual patient data will be analyzed. This study will be published in a peer-reviewed journal.

## 3 Discussion

Endometriosis is one of the most common disorders in reproductive-age women.^[1,2]^ It often results in ERP, and a variety of treatments are reported to mange this condition. However, its efficacy is still not satisfied. Fortunately, as numerous of studies have reported that NMES is effective and safety for the treatment of ERP.^[12–19]^ But, the results are still controversial. Therefore, this planned systematic review will provide a comprehensive summary of the effectiveness and safety of NMES for females who suffer from ERP. The results of this study may supply evidence for clinical practice and health related policy-maker.

## Author contributions

**Conceptualization:** Zi-yang Zhang, Bi-yao Wang, Wei-ting Zhang.

**Data curation:** Jing Wang, Yi-lin Fan, Bi-yao Wang.

**Formal analysis:** Zi-yang Zhang, Jing Wang, Yi-lin Fan, Wei-ting Zhang.

**Funding acquisition:** Wei-ting Zhang.

**Investigation:** Wei-ting Zhang.

**Methodology:** Zi-yang Zhang, Jing Wang, Yi-lin Fan.

**Project administration:** Wei-ting Zhang.

**Resources:** Zi-yang Zhang, Jing Wang, Bi-yao Wang.

**Software:** Zi-yang Zhang, Jing Wang, Yi-lin Fan, Bi-yao Wang.

**Supervision:** Wei-ting Zhang.

**Validation:** Zi-yang Zhang, Yi-lin Fan, Wei-ting Zhang.

**Visualization:** Zi-yang Zhang, Yi-lin Fan, Bi-yao Wang, Wei-ting Zhang.

**Writing – original draft:** Zi-yang Zhang, Jing Wang, Yi-lin Fan, Bi-yao Wang, Wei-ting Zhang.

**Writing – review & editing:** Zi-yang Zhang, Jing Wang, Bi-yao Wang, Wei-ting Zhang.
